# Nurses' actions in response to nursing assistants' observations of signs and symptoms of infections among nursing home residents

**DOI:** 10.1002/nop2.22

**Published:** 2015-08-06

**Authors:** Hanna Allemann, Märta Sund‐Levander

**Affiliations:** ^1^ Faculty of Health SciencesDepartment of Medical and Health SciencesLinköping UniversityLinköpingSweden

**Keywords:** Documentation, nursing assistants, summative content analysis

## Abstract

**Aims:**

To describe what nurses do during episodes of suspected infection in elderly nursing home residents and if these actions are linked to who is initiating an episode and whether the episode is considered an infection or not.

**Design:**

Prospective descriptive study. Data were collected in 2008–2010.

**Methods:**

Summarized and categorized documentation by nursing assistants and nurses was used for summative content analysis.

**Results:**

Nurses' actions seem to be related to who initiated the episode and if the episodes are categorized as ‘non‐infection’, ‘possible infection’ or ‘infection’. Actions could be ‘observation’, ‘screenings’, ‘engaged in waiting’, ‘follow‐ups’, ‘nurse‐prescribed actions’, ‘diagnosing’, ‘contacting the physician’, ‘carrying out an action prescribed by the physician’, ‘contacting an ambulance or arranging an emergency visit to the hospital’ and ‘prescribing screening’. As NAs often initiate episodes of suspected infection by observing changed conditions, it seems important to include the NA in the decision‐making process as these observations could detect possible early signs and symptoms of infections.

## Background

Nursing home residents (NHR) are more likely to be troubled by infections due to general frailty (Yoshikawa 2000, High 2004) and physical impairment (Yoshikawa 2000). The most frequent infections are urinary tract infections (UTI), lower respiratory tract infections (LRTI) and skin and soft tissue infections (SSTI) (Shortliffe & McCue 2002, High *et al*. 2009, Dwyer *et al*. 2013). Signs and symptoms of infection in NHR are often atypical (High *et al*. 2009), resulting in a delay in diagnosis and treatment (Yoshikawa 2000) and increased mortality (Sund‐Levander *et al*. 2003, Arinzon *et al*. 2011). These atypical signs and symptoms consist of absence of fever, weakness, falling, weight loss, physical dysfunction, cognitive decline, delirium and resistive behaviour (Berman *et al*. 1987, Yoshikawa 2000, Gavazzi & Krause 2002, Sund‐Levander *et al*. 2003, High *et al*. 2009, Kovach *et al*. 2010, Arinzon *et al*. 2011). In addition, the presence of comorbidities and changes related to normal ageing and malnourishment might blur the clinical picture (High *et al*. 2009).

In nursing homes (NH), the registered nurse (RN) is responsible for decisions when a resident is unwell, such as whether to contact the physician or not. It has been stated that there are two main types of decisions made by the RN; the decision is either ‘autonomous’ (71%) or ‘collaborative’ (29%). The autonomous self‐directed decisions start and end with the RN. Most of these decisions involve coordinating care or controlling symptoms. When making autonomous consultative decisions, the RN makes the decision and then has the decision confirmed by the physician. Collaborative decisions are about sharing the decision with other professionals, mainly the physician (O'Neill 1997).

In Swedish NH, nursing assistants (NA) provide most of the daily care, supervised by the RN (Kihlgren *et al*. 2003). When the RN makes decisions about how to act, it is therefore likely that she uses information provided by the NA in this process. Hence, NAs can play a key role in detecting and conveying signs and symptoms of infections in the NH (High *et al*. 2009, Tingstrom *et al*. 2010). In a previous study, NAs described early non‐specific signs and symptoms of infection in NHR as ‘is not as usual’, i.e. diffuse behavioural changes and ‘seems to be ill’, i.e. more distinct characteristics and signs of infections. According to the NA, both RNs and physicians pay more attention to the signs described in the ‘seems to be ill’ category (Tingstrom *et al*. 2010).

Jackson and Shafer have investigated the NA's conceptions about flow of information in the nursing home. They have also examined to what extent the NA's understanding of what constitutes an infection corresponds with that of the geriatric nurse practitioner's. The nurse practitioner and NAs agreed that there was an infection present in 4% of the studied cases, whereas they disagreed about a total of 27%. NAs used different terms than nurse practitioners when describing health problems, i.e. ‘cold’ was used by the NAs, whereas nurse practitioners used terms such as ‘asymptomatic urinary tract infection’, ‘fever’, ‘superficial skin infection’ and so forth. Data were interpreted as indicating that vague signs of infections conveyed by NAs to nurse practitioners by, e.g. the broad term ‘cold’, could cause difficulties. If nurse practitioners construe the communicated problem as vague or non‐specific, they may not consider it worth an intervention. It appeared the residents’ outcome depended on NAs’ beliefs, on what they convey to the nurse practitioners and on what they do (Jackson & Schafer 1993).

According to Anderson *et al*., there is reason to believe that there is an insufficient amount of information transferring between the RN and the NA (Anderson *et al*. 2005). In their study, Jackson and Shafer indicate that, if they feel ignored, NAs could be hindered from passing on information to the RN (Jackson & Schafer 1993). Tingström *et al*. (Tingstrom *et al*. 2010) observed that the NAs expressed that they sometimes falsify information, i.e. report that the resident has a temperature of 38°C although it is actually lower, to make the RN take action (Tingstrom *et al*. 2010). Wheeler and Oyebode identified that clear role demarcations were apparent and this caused leaders to feel separate from RNs and RNs from NAs. This was also discussed as being a possible basis for conflicts and could be a cause for staff not passing on information (Wheeler & Oyebode 2010).

Including the NA in the decision‐making process is described as important for quality of care (Anderson *et al*. 2005, Sund‐Levander & Tingstrom 2013). Jackson and Shafer conclude that NAs have a position that often allows them to be the first to spot early signs of infection. If this process is supported, it could result in better quality care (Jackson & Schafer 1993). To the author's knowledge, there are few studies describing how the NA's information affects the RN's decision‐making.

## Aim

The aim of this study was to describe what nurses do during episodes of suspected infection in elderly nursing home residents and to see if these actions can be linked to who is seen as initiating an episode, the nurse or the NA and whether the episode is considered as relating to an infection or not.

## Method

### Design

This study is part of a larger prospective, longitudinal study. The aim of the larger study is to investigate early signs and symptoms and biochemical markers in NHR with suspected infection (Tingstrom *et al*. 2010, Sund‐Levander & Tingstrom 2013). This study has a descriptive design using summative qualitative analysis, inspired by Hsieh and Shannon (Hsieh & Shannon 2005).

### Participants and setting

Residents included in the main study consists of 205 elderly individuals, aged 86 (sd 7) years (66‐101 years), 71% women, living in non‐profit community NH. Sixty‐one per cent were diagnosed with dementia, 56% with chronic heart disease, 37% had had a stroke, 8% had chronic obstructive pulmonary disease and 12% were malnourished (Tingstrom *et al*. 2010). The NHs were situated in two adjacent counties in the south of Sweden.

### Data collection

Data analysed in this study consist of documentation of suspected infection. It was collected from medical, nursing and social care records for the 205 elderly people included in the main study. The documentation was made by NAs, RNs and general practitioners (GP). Data for each individual were collected during approximately 1 year between 2007 and 2010. NAs also had access to a protocol that was developed in the main study and used as support when documenting their observations (Tingstrom *et al*. 2010). The project owner (M SL) and a research assistant summarized documentation by NA, RN and GP concerning signs and symptoms related to infection and merged these into one document.

In a second review, one GP and one geriatric physician independently, based on the summaries, evaluated and classified each documented episode of suspected infection as either ‘infection’, ‘possible infection’ or ‘non‐infection’. Inter‐reliability between the two physicians was tested in a pilot evaluation with 20 selected NHRs. This pilot evaluation of episodes (*n *=* *62 in 20 NHRs), where an NA had suspected infection resulted in 95% full agreement (59/62) in the two physicians’ scoring. In the remaining cases (*n *=* *3), consensus was achieved after discussion. Only these episodes that had been assessed by the physicians and episodes, where it was clear who initiated the episode was used in the final analysis, resulting in data from 141 elderly individuals and 299 episodes of suspected infection.

### Data analysis

The merged documentation was analysed using summative content analysis, which is a flexible method (Hsieh & Shannon 2005) that can be used in both qualitative and quantitative studies (Graneheim & Lundman 2004, Hsieh & Shannon 2005). The method allows pattern detection and interpretation (Morgan 1993), including analysis of discovered patterns through both manifest and latent analysis (Hsieh & Shannon 2005). This method was chosen as there was a large quantity of data (Elo & Kyngas 2008) and there seemed to be a need for both counting and interpretation. The quantification of what the RN does during episodes of suspected infection is referred to as the manifest content (Morgan 1993, Hsieh & Shannon 2005). This quantification was done to detect patterns and these patterns were then interpreted in a further analysis regarded as the latent analysis (Hsieh & Shannon 2005).

Analysis began by reading the material to obtain a sense of the whole (Hsieh & Shannon 2005, Elo & Kyngas 2008). During this first read, some initial notes were made of anything that was unclear in the material, inaccuracies stemming from the merger of the documentation or of what could be interesting to study further. When sorting out the evaluated material, questions about which notes in the documentation could be seen as starting and ending an episode of possible infections were raised. ‘An episode’ concerned documentation by the NA, RN and GP that in time could be tied to one single event of suspected infection. An episode was considered as ending if there was a contact with the GP, who set a diagnosis or started a treatment, i.e. antibiotics. If there was no contact with the GP, or if the contact did not seem as the end, the episode was seen as ending when a blood or urine test was taken. As it was not always clear if the RN noted test results, these were only seen as an end if it was made obvious through documentation. Another possible ending was when the RN discussed a possible cause of a described or diagnosed problem. If there was no such documentation, the episodes were considered as ending when the RN, e.g. documented that the NHR felt better, when the RN did a follow‐up of an NHR admitted to hospital or when there was no more documentation tied to that episode. Sometimes it was difficult to tell what actually ended the episode and in these cases, the end was set according to hierarchy between the above‐stated different possible endings in latent analysis. According to documentation, the RN did nothing in a few cases and in these situations, the end was coded as ‘nothing happens’.

A coding scheme was derived from the documentation itself (Morgan 1993). In this process, episodes were read several times, studying and naming the RN's actions. An action was coded only once per episode, even though it could be that the RN performed the same action more than once. For example, she might do several follow‐ups during a day and night.

### Trustworthiness

Aspects of trustworthiness (Graneheim & Lundman 2004, Hsieh & Shannon 2005) have been considered throughout the analysis, e.g. checking with experts. The excerpts are not quotations, but have been translated in such a manner that meaning shall not be lost. Another way to understand and be able to validate the data is to get to know the context where it was collected, as qualitative content analysis concerns the interpretation of a text and the text is depending on context (Burnard 1991, Krippendorff 2013 2013). Therefore, the main author (H A) visited one NH on two separate occasions. The project owner and co‐author (M SL) is highly familiar with the context where the study took place.

For validation, the project owner (M SL) went through the codes in the code scheme and assessed them to be usable in this context. In addition, the research group consisting of RNs, physicians and specialists in immunology discussed the evaluation of the quotations as no infection, possible infection or infection.

## Ethical considerations

The prospective, longitudinal main study was approved by the Regional Ethical Review Board. That approval also includes this study. Ethical guidelines for nursing research in the Nordic countries and the Declaration of Helsinki were considered.

## Findings

The code scheme (Table 1) comprising the 11 codes illustrates the RN's actions in episodes where an infection could be suspected. According to what could be deduced from the documentation, the NA initiated an episode more often than the RN. During analysis, the episodes where it was difficult to tell whether the nurse or NA initiated was excluded (Table 2). When the NA was seen as the initiator, the three most common nursing actions that emerged in the ‘non‐infection’ category were: ‘observation’, ‘nothing happens’ and ‘follow‐up’. In the ‘possible infection’ category, these actions seemed to be: ‘observation’, ‘contacting the physician’ and ‘nothing happens’ and in the ‘infection’ category these were: ‘observation’, ‘contacting the physician’ and ‘follow‐up’. When the nurse was seen as the initiator, the three most common nursing actions that emerged in the ‘non‐infection’ category were: ‘observation’, ‘follow‐up’ and ‘screening’. In the ‘possible infection’ category, these actions seemed to be: ‘observation’, ‘contacting the physician’ and ‘screening’ and in the ‘infection’ category these were: ‘observation’, ‘contacting the physician’ and ‘follow‐up’ (Table 3).

**Table 1:** Code scheme

Observation	Was used for when the nurse observed and also documented something. This could be something that the nurse discovered by herself or something that the nursing assistant drew her attention to.
Screening	Was used when the nurse, e.g. took a blood test, blood pressure or urine test.
Engage in waiting	Was used when the intervention was to wait and this was clearly stated in the documentation. Could be for a shorter or a longer period of time.
Follow‐up	Was used when the nurse followed up on a previously described observation. It was also used when the nurse checked for a test result or did a follow‐up on a resident who was admitted to hospital.
Nurse prescribed action	Was used when the nurse, e.g. attended to catheters, handed out medicines or cooled down a warm room. Was seen as being prescribed by the nurse herself or as a part of “standard care”.
Diagnosing	Was used when the nurse documented a diagnosis or a probable cause of a problem. This includes establishing that an elderly person was tired and that the cause could be a side effect of a certain medicine. It could also be when the nurse established a cold.
Contacting the physician	Was used for rounds or for when the nurse initiated a contact herself outside scheduled time.
Carrying out an action prescribed by the physician	Was used when the nurse did what the physician prescribed.
Nothing happens	Was used when the nurse did not document anything, even though the nursing assistant seemed concerned for an elderly person. It was also used when there seemed to be an interlude in the documentation during an episode. This was only coded when nothing happens for a whole day and night.
Contacting an ambulance or arranging an emergency visit to the hospital	Was used when the elderly person was acutely sent to hospital.
Prescribing screening	Was used when the nurse prescribed a screening for later on. This could include contacting the physician the next day, taking a temperature in the evening or measuring a blood pressure the next morning.

**Table 2:** Who is seen as initiating an episode

	Non‐infection	Possible infection	Infection
Initiated by			
Nursing assistant	42 (57%)	59 (49%)	83 (43%)
Nurse	20 (27%)	43 (36%)	52 (27%)
Nurse or nursing assistant	12 (16%)	18 (15%)	55 (29%)
Nurse, nursing assistant or physician	0 (0%)	0 (0%)	2 (1%)
Sum	74	120	192

**Table 3:** In percent, frequencies of actions in the different categories considering who is seen as initiating the episode

Nurses' actions	Non‐infection		Possible infection		Infection	
	Nursing assistant 42 episodes	Nurse 20 episodes	Nursing assistant 59 episodes	Nurse 43 episodes	Nursing assistant 83 episodes	Nurse 52 episodes
Engage in waiting	7%	20%	5%	5%	11%	4%
Screening	38%	70%	41%	53%	54%	58%
Nurse prescribed action	17%	10%	17%	12%	42%	35%
Observation	79%	100%	97%	100%	99%	98%
Follow‐up	64%	80%	46%	37%	67%	67%
Contacting the physician	33%	55%	83%	95%	87%	79%
Carrying out an action prescribed by the physician	5%	10%	8%	2%	1%	2%
Nothing happens	69%	35%	61%	33%	42%	27%
Contacting the ambulance or arranging acute visit to the hospital	0%	0%	3%	2%	10%	10%
Prescribing screening	21%	35%	15%	23%	37%	27%
Diagnosing	10%	35%	19%	21%	20%	38%

An observation was seen as starting an episode in approximately 72%, irrespective of whether the NA or RN was the initiator. Of episodes that did not start with an observation, a large proportion started with ‘nothing happens*’,* i.e. when the NA noted something that either did not reach the RN, or that the RN did not document.

A common end of an episode was when the RN ‘made contact with the physician’. Contacting the physician seemed to end 73% of the episodes, irrespective of category or initiator. It was more common as an end in the categories ‘possible infection’ and ‘infection’ (Table 4).

**Table 4:** Contact with the physician. In how many episodes of suspected infection did the episode end with contacting the physician?

	Initiator	Number and %, latent analysis
Non‐infection	Nursing assistant	13/42 (31%)
	Nurse	11/20 (55%)
Possible infection	Nursing assistant	46/59 (78%)
	Nurse	40/43 (93%)
Infection	Nursing assistant	67/83 (81%)
	Nurse	41/52 (79%)

The excerpt below from the ‘infection’ category illustrates an observation by the NA ending with the RN contacting the GP:

A temperature this morning…has difficulty breathing and is very tired and affected. Contacts the physician, antibiotics…prescribed. Blood tests for the study taken…Even more difficulty breathing. Taken to hospital by ambulance.

In episodes where the GP was not contacted, ‘follow‐ups’ were coded as the most common ending. In the ‘possible infection’ and ‘non‐infection’ category*,* ‘follow‐ups’ involved checking up on a previous observation or on test results. In the ‘infection’ category, most of the ‘follow‐ups’ involved checking up on an NHR admitted to hospital.

In total 12 episodes, the GP was contacted, although not ending the episode. In these episodes, a ‘follow‐up’ was made on admission to hospital where the actual diagnosis was set.

In some episodes, mainly in the ‘non‐infection’ category, the end was ‘nothing happens’*,* while in the ‘infection’ category, the end was coded with ‘diagnosing’*,* mostly as a cold, by the RN. A few episodes ended with either an ‘observation’, ‘RN prescribed action’ or a ‘screening’.

*‘*Nothing happens’ was coded in 45% of the episodes, i.e. the RN did not act at all during a whole day and night. This was more common when the NA was the initiator, illustrated in the following excerpt:

RN, day 1 Has complained about stomach pain to personnel, does not know when she last had a bowel movement

RN, day 4 Still does not know if she has had a bowel movement

RN, day 6 Has had a bowel movement? Not swollen or hard when palpating the stomach.

RN, day 7 Does not seem to be affected today. Urine test taken. Awaiting contacting the physician as NN does not have symptoms of a UTI.

NA day 8 Loss of appetite, does not want to eat, burning/thick urine, watery eyes, disorderly, messy, pain.

RN, day 8 Still unclear whether or not she has had a bowel movement. Is not affected. No stomach pain or nausea.

NA, day 9 Feeble, eats less, pain, worry, confusion, burning sensation from the urinary tract.

RN, day 10 Is more confused, has had a bowel movement. Urine test is positive for white blood cell, negative nitrite. Is very tired. Pain in the lower parts of the stomach. Is prescribed antibiotics.

The following excerpt from the ‘possible infection’ category, illustrates when ‘nothing happens’ initiates the episode:

NA, day 1 Urine test shows maximum on everything.

RN, day 4 An itch in the genitals. Urine status positive. Will be discussed at rounds today… is prescribed antibiotics.

An excerpt from the ‘infection’ category, when ‘nothing happens’ is coded both as initiating and appearing during an episode:

NA, day 1: Complained of burning sensation when urinating
NA, day 5: Urine test taken. Nurse contacted. New urine test for urine culture tomorrow
RN, day 5: Dry, cool skin. Urine test taken, positive nitrite. More worried than usual. Urine test for urine culture tomorrow. Not long since NN was treated with antibiotics
RN, day 10: Urine culture shows bacteria, among others pseudomonas. Is prescribed antibiotics.

In 12 episodes, mainly from the non‐infection category, ‘nothing happens’ was the only event, i.e. both starting and ending an episode. In most of these episodes, a temperature or a urine sample was taken by the NAs. In almost all cases, the episodes seemed to last for one day only. An excerpt illustrates when the RN was not involved:

Food intake, general signs of disease, symptoms from the airways, tired, bodily pain.

Also, a temperature just below 38°C was noted.

When ‘nothing happens’ was seen as starting an episode, it seems that the RN was sometimes initiated already from the first day of the episode, even though the nurse did not document.

Took a urine test. Contacted the nurse: await.

## Discussion

The challenge with detecting and diagnosing infections in frail elderly individuals due to non‐specific signs and symptoms (High *et al*. 2009), is a well‐known clinical problem. These findings indicate some differences in what the RN does depending on infection status evaluated in the study. Although the RN is responsible for assessment of the individuals’ condition and deciding on whether or not to take action, she needs to cooperate with other professions in the decision‐making process, especially the NA (High *et al*. 2009, Tingstrom *et al*. 2010). As almost half of the episodes evaluated as possible infection or infection were initiated by NA, it is significant that the RN consider the information as valid in the decision process. To avoid falsification of information (Tingstrom *et al*. 2010), it is important that NAs are taught how to express and convey early signs and symptoms of infections in the nursing home setting (Jackson & Schafer 1993).

The present results show that the actions the RN performed varied depending on who initiated the episode. However, it might be that the actions taken depends more on the possible presence of an infection than on the initiator. Not surprisingly, looking at data from the perspective of who is seen as the initiator and how the episode is categorized with regard to infection, it appears that an ‘observation’ is common, no matter who is initiating or how the episode is categorized.

It appears that ‘contacting the physician’ is not as common in episodes assessed as ‘non‐infection’ as in episodes of ‘possible infection or infection’. This could be interpreted as a correct assessment by the RN, or in some cases, a correct assessment by the NA, i.e. the NA describe changed condition in terms of ‘seems to be ill’ rather than ‘is not as usual’ (Tingstrom *et al*. 2010). This process could also be seen as the RN making an autonomous decision (O'Neill 1997).

‘Nothing happens’ is most common when the NA is the initiator in the ‘non‐infection’ and ‘possible infection’ categories. When an infection is assessed, it appears that it does not matter who is the initiator, the most common actions are: ‘observation’, ‘contacting the physician’ and ‘follow‐up’. This could be due to how signs and symptoms are communicated by the NA (Berman *et al*. 1987, Yoshikawa 2000, Gavazzi & Krause 2002, Sund‐Levander *et al*. 2003, High *et al*. 2009, Kovach *et al*. 2010, Arinzon *et al*. 2011), the blur of comorbidities and changes related to normal ageing and malnourishment (High *et al*. 2009). The RN contacting the physician could be seen as either an autonomous consultative or a collaborative decision (O'Neill 1997). Contacting the physician is also very common in the ‘possible infection’ category, i.e. documentation and test results not supporting or dismissing an infection. A question raised is if this mirrors the RNs’ difficulty assessing clinical sign and symptoms and therefore the need to consult the physician. The fact that admissions and acute visits to hospital principally only happened in the ‘infection’ category supports that RN's role in decisions about whether to transfer a resident to hospital are justifiable (Jablonski *et al*. 2007).

The most common start of an episode is an ‘observation’. When this is not the case, the episode most commonly starts with ‘nothing happens’. Especially when the NA was coded as initiating an episode in the ‘non‐infection’ category, ‘nothing happens’ both started and ended an episode. It could be that the NA themselves observed something in the NHR, took a test and decided not take their suspicion further (Sund‐Levander & Tingstrom 2013), supporting that the NA's conclusions were correct. However, this does not say anything about whether the NA is clear or comfortable about this, e.g. a negative urine test result, not confirming their suspicion, could cause frustration and also affect the information passed on to the RN (Sund‐Levander & Tingstrom 2013). NAs has reported that they are experts when it comes to the residents, but that they feel that their knowledge is not taken advantage of (Carpenter & Thompson 2008). However, in this study, it did not always seem that the test result was negative. For example, an elevated temperature was sometimes documented, seemingly without any further actions. It can, therefore, not be ruled out that the documentation in these episodes is not fully representative of what actually happened.

It is described that RNs in NH use an analytical approach to decision‐making (Lauri & Salantera 1998, Lauri *et al*. 2001) and that this decision‐making could be influenced by the RN trying to satisfy all sides (Lopez 2009), the RN feeling safe in her role (Kihlgren *et al*. 2003) and well‐functioning cooperation with others (Kihlgren *et al*. 2003, Sikma 2006). To the authors’ knowledge, there are few studies directly discussing how the NA influences the RN's decision‐making and how the RN makes use of this information in deciding what to do next. It is possible that the information from the NA affects, e.g. the understanding of facts (Mahon 2010) necessary in decision‐making. When the information reaches the RN, they still need to assess it and decide on further actions. This process could be obstructed by the fact that the NA does not necessarily use the same language as the RN (Jackson & Schafer 1993) and that the NA does not always feel safe in conveying suspicions to the RN (Sund‐Levander & Tingstrom 2013). Too little information passing between the RN and NA in NH could deteriorate quality of care (Anderson *et al*. 2005). Furthermore, the information passed on needs to be of good quality (Preuss 2003).

## Conclusion

When assessing signs and symptoms of infection in NHR, there is a relationship between what the RN does, who is seen as initiating an episode and if the episodes are categorized as ‘non‐infection’, ‘possible infection’ or ‘infection’. As NAs often initiate episodes of suspected infection by observing changed conditions, it is important to include the NA in the decision‐making process as these observations could detect possible early signs and symptoms of infections. The result indicates good care in that the physician is not contacted as much in the ‘non‐infection’ category, that calling for an ambulance or arranging acute care is mostly seen in the ‘infection’ category and that most of the episodes that both start and end with the NA are found in the ‘non‐infection’ category. The patterns discovered in this study need to be examined further to validate their implications. For example, it could be of value to study how RNs use information about early signs of infections in NHR given by the NA in real time in NH, e.g. in an ethnographic study.

### Limitations

Data itself can be seen as a weakness as it is difficult to tell whether the written documentation is the same as what actually happened. The documentation is most likely not fully representative, taking into consideration factors such as verbal reports, communication patterns and relationships between the RN and NA. However, this study is interpreted as showing patterns and constituting the basis for further study. Another potential weakness is that the documentation was not assembled by the author. However, it was clarified to the author that the summarization and shortening of documentation was done while keeping in mind that these should not affect the actual meaning of the data. Questions about inaccuracies, the summarization and coding made by the physicians were raised and answered in the project group as they appeared throughout the analysis process. To minimize the effects of these shortcomings, start points were checked with the project owner and end points were checked several times to make sure that that episodes were coded according to the same standard. A few episodes were removed as it was too difficult to determine the start and end. As these were few, discovered patterns should not be affected.

## Conflict of interest

The authors declare no conflict of interest in performing the study or preparing the manuscript.

## Author contributions

All authors have agreed on the final version and meet at least one of the following criteria [recommended by the ICMJE (http://www.icmje.org/recommendations/)]:

- substantial contributions to conception and design, acquisition of data or analysis and interpretation of data;
- drafting the article or revising it critically for important intellectual content.
